# Efficacy, safety and tolerance of imidocarb dipropionate *versus* atovaquone or buparvaquone plus azithromycin used to treat sick dogs naturally infected with the *Babesia microti*-like piroplasm

**DOI:** 10.1186/s13071-017-2049-0

**Published:** 2017-03-13

**Authors:** Rocío Checa, Ana Montoya, Nieves Ortega, José Luis González-Fraga, Adrián Bartolomé, Rosa Gálvez, Valentina Marino, Guadalupe Miró

**Affiliations:** 1 Department of Animal Health, Veterinary Faculty, Universidad Complutense de Madrid, Avenida Puerta de Hierro s/n, 28040 Madrid, Spain; 2 Xarope Veterinary Centre, C/Santa Lucía 42, 15145 Laracha, Coruña Spain; 3 Gran Vía Veterinary Centre, C/Gran Vía 194, 15102 Carballo, Coruña Spain

**Keywords:** *Babesia microti*-like piroplasm, Canine piroplasmosis, Therapeutic efficacy, Imidocarb dipropionate, Azithromycin, Atovaquone, Buparvaquone

## Abstract

**Background:**

Piroplasmosis caused by the *Babesia microti*-like piroplasm (Bml) is increasingly being detected in dogs in Europe. Sick dogs show acute disease with severe anaemia associated with thrombocytopenia with a poor response to current available drugs. This study assesses the safety and tolerance of three treatments and compares their efficacy over a full year of follow up in dogs naturally infected with Bml.

**Methods:**

Fifty-nine dogs naturally infected with Bml were randomly assigned to a treatment group: imidocarb dipropionate (5 mg/kg SC, 2 doses 14 d apart) (IMI); atovaquone (13.3 mg/kg PO q 8 h, 10 d)/azithromycin (10 mg/kg PO q 24 h, 10 d) (ATO); or buparvaquone (5 mg/kg IM, 2 d apart)/azithromycin (same dosage) (BUP). Before and after treatment (days 15, 45, 90 and 360), all dogs underwent a physical exam, blood tests and parasite detection (blood cytology and PCR). Clinical efficacy was assessed by grading 24 clinical and 8 clinicopathological signs from low to high severity.

**Results:**

Before treatment, most dogs had severe regenerative anaemia (88.13%) and thrombocytopenia (71.4%). On treatment Day 45, clinical signs were mostly reduced in all dogs, and by Day 90, practically all dogs under the ATO or BUP regimen were clinically healthy (76.4 and 88%, respectively). Highest percentage reductions in laboratory abnormalities (82.04%) were detected in animals treated with ATO. Over the year, clinical relapse of Bml was observed in 8 dogs (8/17) treated with IMI. However, on Day 360, these animals had recovered clinically, though clinicopathological abnormalities were still present in some of them. Parasitaemia was PCR-confirmed on Days 90 and 360 in 47.05 and 50% of dogs treated with ATO, 68 and 60.08% with BUP, and 94.1 and 73.3% with IMI, respectively. Even after 360 days, 13.3% of the dogs treated with IMI returned a positive blood cytology result.

**Conclusions:**

IMI showed the worse clinical and parasitological, efficacy such that its use to treat Bml infection in dogs is not recommended. The treatments ATO and BUP showed better efficacy, though they were still incapable to completely eliminate PCR-proven infection at the recommended dose. All three treatments showed good tolerance and safety with scarce adverse events observed.

## Background

Canine babesiosis is a serious tick-borne disease caused by several species of the protozoan genus *Babesia* [1, 2]. Historically, *Babesia* infection in dogs was identified according to the morphological features of the parasite within the animal’s red blood cells. Based on relative size, these parasites are broadly divided into two groups, large and small piroplasms [2]. However, modern molecular techniques have resulted in the identification of three genetically distinct small *Babesia* parasites in dogs: *Babesia gibsoni*, *Babesia conradae* and the *Babesia microti*-like isolate. This last species has been recently reported as “Babesia vulpes” by Baneth et al.[3] and also described as “Babesia Spanish dog isolate", “Babesia (Theileria) annae”, or *Babesia* cf. *microti* [1].

The *Babesia microti*-like isolate (Bml) is a small piroplasm first described in north-western Spain (as “Babesia (Theileria) annae” or *Babesia* cf. *microti*) where Bml infection in dogs is now considered endemic [4, 5]. Sick dogs feature an acute disease with clinical signs such as pale mucous membranes, anorexia, apathy, fever, haematuria, splenomegaly, weight loss, regenerative macrocytic hypochromic anaemia and thrombocytopenia [5]. Bml infection is recognized as a serious disease because of its poor response to current piroplasmosis treatments (imidocarb diproiponate) [6].

More recently, the antiprotozoan hydroxynaphtoquinone, atovaquone, has proved effective against *B. divergens* and *B. microti* infection in cows and humans [7, 8]. In combination with the antibiotic azithromycin, atovaquone eliminates *B. microti* parasitaemia in both humans and hamsters, while neither drug alone eliminates *Babesia* parasitaemia [9, 10]. Other piroplasms including *B. gibsoni*, *B. conradae* and *Cytauxzoon felis* have shown a response to combined atovaquone/azithromycin treatment [11–13]. This drug combination is the only treatment able to reduce *B. gibsoni* parasitaemia below the PCR limit of detection [12], and also seems effective for the treatment of acute and chronic piroplasmosis caused by *B. conradae* [13]. The mechanism of action of atovaquone against protozoans is through cytochrome *b* and electron transport inhibition [14]. The most commonly used dosing regimen is 13.5 mg/kg of atovaquone administered orally (PO) every 8 h with fatty food (to maximize drug absorption) in combination with azithromycin (at a dose of 10 mg/kg PO) for ten days [1, 12].

The hydroxynaphthoquinone buparvaquone was developed in the 1980s. Buparvaquone has been extensively tested for veterinary use against bovine theileriosis [15, 16]. Some authors have also reported the use of buparvaquone for the treatment of equine piroplasmosis caused by *Babesia equi* [17, 18]. Buparvaquone has also shown activity against other protozoan parasites such *Plasmodium* spp*.* [19], *Leishmania* spp. [20] or *Neospora* spp. [21, 22]. Although the mechanism of action of this drug has not been fully elucidated, there are indications of its selective toxicity through inhibition of parasite respiratory systems as postulated for *Theileria* spp., *Plasmodium* spp., and *Eimeria* spp. [19]. Today, buparvaquone is commercially available for use against theileriosis in cattle in some African countries.

To date, however, no drug has proved capable of clearing Bml infection in dogs. Imidocarb dipropionate remains the first choice treatment against large *Babesia* species infection, but when used to treat small species infection, clinical relapses are very frequents [7]. Notwithstanding, this drug is often used to treat Bml infection in dogs in Europe because it is the most accessible and cheapest treatment available. The present study compares the efficacy of three anti-*Babesia* treatments (imidocarb dipropionate, and the combination treatments atovaquone/azithromycin and buparvaquone/azithromycin) over a full year of follow up in dogs naturally infected with Bml. To the best of our knowledge, this is the first report of the use of buparvaquone to treat sick dogs infected with Bml.

## Methods

### Study design

Sixty dogs naturally infected with Bml from seven veterinary practices in NW Spain (five in Galicia and two in Asturias) were initially enrolled in this multicentre, randomized not blinded trial. Before treatment (Day 0), all participating dogs were subjected to a clinical examination and blood collection by cephalic venipuncture for screening for vector-borne diseases, complete blood counts (CBC), biochemical tests, and blood smear and PCR to confirm Bml infection. The study was carried out in accordance with the International Guiding Principles for Biomedical Research Involving Animals issued by the Council for International Organizations of Medical Sciences. Owner consent was obtained in all cases.

Criteria for inclusion were to present: (i) at least three clinical signs consistent with canine piroplasmosis: apathy, poor appetite to anorexia, pale mucous membranes, jaundice, pigmeturia or pigmented faeces (indicating bilirubin excretion), haematuria and fever; and (ii) positive blood smear confirmed by nested PCR and sequencing.

Dogs with Bml infection were excluded if they fulfilled at least one of the following exclusion criteria: (i) pregnant or lactating females; (ii) any vector-borne disease concomitant infections including *B. canis*, *L. infantum*, *E. canis*, *Anaplasma* spp., and *Leptospira* spp.; (iii) severe kidney, liver, or heart failure; and (iv) treatment with antibiotics, antifungals, corticosteroids and/or a specific anti-*Babesia* agent within 60 days.

During the course of the study, dogs were withdrawn if they met any of the following criteria: (i) use of different treatment to that assigned; (ii) adverse events requiring interruption of treatment or follow-up; and (iii) a secondary infection or illness requiring a change in treatment.

### Screening for vector-borne diseases

Before enrollment, blood samples were collected from each dog to screen for the most prevalent companion vector-borne diseases (CVBD) in Spain [23] by different techniques: other *Babesia* species (PCR and sequencing) [23], *Leishmania infantum* and *Ehrlichia canis* (immunofluoresent antibody test, in-house), *Anaplasma* spp. and *Leptospira* spp. (SNAP® 4DX; SNAP® Lepto Test -IDEXX Laboratories).

### Treatment groups

Each dog was randomly assigned to a treatment group: 17 dogs were treated with imidocarb dipropionate (5 mg/kg SC twice 15 days apart) (IMI group); 17 dogs were treated with atovaquone (13.5 mg/kg PO TID/10 days) plus azithromycin (10 mg/kg PO SID/10 d) always administrated with fatty food (ATO group); and 26 dogs were treated with buparvaquone (5 mg/kg IM twice 48 h apart) plus azithromycin (10 mg/kg PO SID/10 d) (BUP group). All included dogs were treated with acaricides against ticks throughout the study on a regular basis.

If, during the study, a dog showed clinical relapse despite treatment indicated by parasitaemia (blood smear) and/or PCR, the animal was subjected to the same treatment protocol as before.

Atovaquone and azithromycin were used out of label since these were not available for veterinary use in Spain at the time of our study. Buparvaquone was imported from Egypt (under authorization by the Spanish Medication and Medical Products Agency) where it is licensed to treat bovine theileriosis.

### Sample collection

All dogs underwent a thorough physical exam, blood counts and biochemical profiling along with parasite detection after treatment (Days 15, 45, 90 and 360). From each dog, a 4.5 ml blood sample was obtained by cephalic venipuncture and 1.5 ml of the collected blood placed in two EDTA tubes: a 1 ml tube for CBC and blood smears and a 0.5 ml tube for Bml detection by genomic DNA isolation and nested PCR. The remaining 3 ml of blood were placed in tubes without anticoagulant for biochemical profiles and serological testing. All blood samples were kept at 4 °C until processing.

### Treatment efficacy

#### Physical examination

Each dog was scored by the same veterinarian before (Day 0) and after treatment (Days 15, 45, 90 and 360) for 23 clinical signs using a categorized scoring system from 0 to 3 (from low to high severity) to obtain an over-all clinical score (maximum score of 63) (Table 1 adapted from Miró et al. [24]). The clinical response to treatment was assessed by examining changes produced in clinical scores over time as score percentage reductions (PR) calculated as described by Hernández et al. [25].

**Table 1:** Score system used to grade 23 clinical signs in dogs with Bml infection (maximum score = 63)

Condition and affected organ	Clinical sign	Severity grade			
		0	1	2	3
General	Appetite	Normal	Reduced	Anorexia	
	Asthenia	Absence	Reduced	Mild	Prostration
	Fever (≥ 39.5 °C)	Absence	39.5–40 °C	> 40	
	Polyuria/Polydipsia	Absence	Drinks less than twice normal amount	Drinks from 2 to 4 times normal amount	Drinks more than 4 times normal amount
Body condition	Weight loss	Absence	Reduced (< 10%)	Mild (10–20%)	Severe (> 20%)
Mononuclear phagocyte system	Enlarged lymph nodes	Absence	Localized (1 or 2 enlarged nodes)	Localized (more than 2 enlarged nodes)	Generalized
	Splenomegaly	Absence	–	Presence	–
	Hepatomegaly	Absence	–	Presence	–
Mucosae	Pale mucous membranes	Absence	Light (membranes pale)	Mild	Severe (membranes white)
	Jaundice	Absence	Light	Mild	Severe (yellow mucous membranes)
	Epixtasis	Absence	Occasional	Frequent	Permanent
	Haematuria	Absence	Light	Mild	Severe
	Dark urine (bilirubin excretion)	Absence	Light	Mild	Severe (dark urine)
	Vomiting	Absence	Occasional	Frequent	Haematemesis
	Diarrhoea	Absence	Occasional	Frequent	Haematochezia
	Melena	Absence		Occasional	Frequent
	Constipation	Absence	Occasional	Frequent	Severe tenesmus
	Faeces	Normal			Dark (bilirubin excretion)
Musculo-skeletal system	Limp	Absence	Light	Moderate	Severe
Cardiac and respiratory System	Tachycardia	Absence	–	Presence	–
	Tachypnea	Absence	–	Presence	–
Skin	Petequias	Absence	≤ 10% of body surface	10–25% of body surface	≥ 25% of body surface
	Equimosis	Absence	≤ 10% of body surface	10–25% of body surface	≥ 25% of body surface

#### Clinicopathological signs

Dogs were also scored for 8 clinicopathological abnormalities (CBC and biochemical profile) using a categorized scoring system from 0 to 2 (maximum score of 12) (Table 2). Percentage reductions in scores over time were also calculated. Complete blood counts included leukocyte count, red blood cell count (RBC), haemoglobin concentration, haematocrit, red cell distribution width (RDW), mean corpuscular volume (MCV), mean corpuscular haemoglobin (MCH), mean corpuscular haemoglobin concentration (MCHC) and platelet count. Biochemical profiles included total serum protein, urea, creatinine, aspartate aminotransferase (AST), alanine aminotransferase (ALT) and symmetric dimethylarginine (SDMA) (only determined in 9 dogs due to the recent availability of this test on the market).

**Table 2:** Score system used to grade clinicopathological abnormalities in dogs with Bml infection (maximum sore = 12)

Variable		Severity grade		
		0	1	2
CBC	Haematocrit/haemoglobin	Normal	Regenerative	Non regenerative
	Leukocytes	Normal	Leukocytosis	Leukopenia
	Platelets	Normal	–	Thrombocytopenia
Biochemical profile	Total serum protein	Normal	Proteinaemia	Hypoproteinaemia
	Urea	Normal	Elevated	–
	Creatinine	Normal	Elevated	–
	ALT	Normal	Elevated	–
	AST	Normal	Elevated	–

### Parasitological follow up

#### Blood cytology

Thin blood smears were examined by light microscopy (LM) to detect intraerythrocytic forms consistent with small *Babesia* merozoites. The smears were air-dried, fixed in absolute methanol for 5 min, stained using 20% Giemsa and then microscopically examined using a 1000× magnification objective under immersion oil. The level of parasitaemia was subjectively defined as low, moderate or high [10]. All smears were examined by the same technician.

#### DNA isolation, nested PCR and sequencing

Genomic DNA was isolated from peripheral whole blood (200 μl) before (Day 0) and after treatment (Days 90 and 360) using the QIAamp® DNA blood mini kit (Qiagen, Barcelona, Spain) following the manufacturer’s instructions. The extracted DNA was eluted in sterilized water (200 μl) and stored at -20 °C until further use.

On Days 0, 90 and 360, blood DNA was detected by nested amplification of the 18S rRNA gene fragment. On Day 0 Blood-DNA was screened for piroplasms using PCR assays including a shorter nested PCR (850 bp; primers BT F1/R1 followed by BT F2/R2) [26]. PCR products corresponding to the expected length were purified (15 μl) using a QIAquick Purification Kit (Qiagen) as described by the manufacturer and sequenced at the Genomic sequencing service (UCM) using an ABI Prism 3730 (Applied Biosystems).

Sequence chromatogram files were analyzed by Chromas 2.1.1, and imported into BioEdit v7.0.5, for editing, assembly and alignments. The sequences obtained during the present study were aligned to sequences available from GenBank using Clustal W and compared whit the additional piroplasms sequences available from GenBank using BLAST program (https://blast.ncbi.nlm.nih.gov/Blast.cgi) to determinate percentage identity of the generated sequences against published sequences.

The nested PCR procedure used during follow-up has been recently validated for the detection of Bml by nested amplification of the 18S rRNA gene fragment using the universal *Babesia-Theileria* primers BT1-F (5'-GGT TGA TCC TGC CAG TAG T-3') and BTH-1R (5'-TTG CGA CCA TAC TCC CCC CA-3') [27] for primary, and the *B. microti*-like isolate specific primers BTFox1F (5'-AGT TAT AAG CTT TTA TAC AGC-3') and BTFox1R (5'-CAC TCT AGT TTT CTC AAA GTA AAA TA-3') for the second amplification round obtained a fragment of 655 bp [28].

The reaction mixture (adapted from Bartley et al. [28]) and amplification conditions were as follows: 2 μl of extracted DNA was added to a 23 μl volume of reaction mixture containing 0.75 units of Tth Plus DNA polymerase 5U/μl (Biotools B&M Labs., Madrid, Spain), 200 μM (each) deoxyribonucleotides (dATP, dTTP, dGTP, dCTP) (Biotools B&M Labs), 10 pmol of each primer (Thermo Fisher Scientific, Paisley, Scotland), 2.5 μl 10× PCR buffer and 1.5 mM MgCl_2_ (Biotools B&M Labs). Negative (2 μl dH_2_O) and positive (the *B. microti*-like isolate DNA) control samples were included in each PCR assay.

PCR products were run on a 1.5% agarose gel containing SYBR Safe Gel Stain (Invitrogen, USA), and visualized with a dark reader trans-illuminator (Clare Chemical, USA).

### Statistical analysis

Results were analyzed using the statistics package SAS version 9.4. Each treatment group was independently compared with the other two. Mean differences in outcome variables between the different days post-treatment were compared by repeated measures ANOVA. This model included group, time-point, and the interaction time by treatment as fixed effects, and subjects (dogs) as the random effect. Since the effect of time by treatment was found globally significant in the ANOVA, the non-parametric Kruskal-Wallis test was used to compare groups at each time point. Since the Kruskal-Wallis test was significant in each group, *post-hoc* pairwise Wilcoxon signed rank tests were performed to establish differences between groups between time-points. For categorical variables, the Chi-square test was used. Significance was set at *P* ≤ 0.05.

## Results

Of the 60 dogs initially enrolled, one dog treated with buparvaquone/azithromycin (BUP) suffered clinical relapse with azotaemia on Day 90. Despite further treatment with atovaquone/azithromycin (ATO), this dog died due to renal failure. Of the final study population of 59 dogs, several dogs were lost to follow up during the course of the study: eight on D15 (6 in IMI, 2 in BUP), seven on D45 (3 IMI, 3 BUP, 1 ATO) and eight on D360 (2 IMI, 3 BUP, 3 ATO). In all but one case, the reason for these losses was that owners failed to return for the re-visits. One of the dogs lost to follow up on D360 was run over by a car.

All included dogs were positive by nested PCR and sequenced on Day 0. When we compared our 60 sequencing results with existing GenBank entries, the sequences obtained were identified as *B. microti-*like isolate in BLAST searches. All the sequences obtained were 99–100% identical to several isolates of “Babesia annae” (“Babesia annae”, “Theileria annae”, “Theileria annae isolate Dog#8”, accession numbers KT580785, JX679165 and JX454779, respectively). No other *Babesia* species were identified in the dog blood samples.

### Comparison of treatment groups at baseline

Of the 59 participating dogs with Bml, 29 were female and 30 male; mean age was 3.23 years (95% CI: 2.54–3.91); mean body weight was 16.58 kg (95% CI: 13.99–19.17) and 39 were hunting dogs and 20 companion dogs. Animal distributions across the three treatment groups were homogenous and there were no significant differences in relation to sex (*χ* ^2^ = 1.082, *df* = 2, *P* = 0.58), lifestyle (*χ* ^2^ = 1.267, *df* = 2, *P* = 0.53) or age (*F*_(2,53)_ = 2.840, *P* = 0.06) among the three groups (Tables 3 and 4).

**Table 3:** Baseline data (D0) recorded in dogs of the three treatment groups. Categorical variables

Variable		ATO	BUP	IMI	*P-*value
Sex (*n*)	Female	10	12	7	0.58
	Male	7	13	10	
Lifestyle (*n*)	Hunting	13	16	10	0.53
	Companion	4	9	7

**Table 4:** Baseline data (D0) recorded in dogs of the three treatment groups. Continuous variables

Variable	Treatment group	Dogs (*n*)	Mean ± SD	95% CI	Min-Max	*P-*value
Age (years)	ATO	17	4.26 ± 2.56	2.94–5.58	0.25–9	0.06
	BUP	24	3.16 ± 2.71	2.01–4.30	0.16–10	
	IMI	15	2.17 ± 1.94	1.09–3.24	0.25–6	
Body weight (kg)	ATO	17	15.23 ± 9.80	10.28–20.37	6.50–36	0.78
	BUP	25	16.67 ± 10.34	12.40–20.90	2.40–52	
	IMI	17	17.72 ± 9.88	12.60–22.80	5.3–40	
Temperature (°C)	ATO	17	38.70 ± 0.84	38.20–39.13	36.5–40.2	0.2
	BUP	25	39.00 ± 0.74	38.70–39.30	37–40.6	
	IMI	17	39.10 ± 0.60	38.70–39.40	38–40	
Clinical score D0	ATO	17	13.00 ± 7.82	8.90–17.02	3–27	0.85
Clinical signs (points)	BUP	25	12.40 ± 6.20	9.80–14.90	3–27	
	IMI	17	11.76 ± 5.17	9.10–14.42	7–24	
Clinical score D0	ATO	17	3.23 ± 1.70	2.30–4.15	1–7	0.4
Clinicopathological abnormalities (points)	BUP	25	2.70 ± 1.30	2.16–3.27	1–5	
	IMI	17	2.70 ± 0.83	2.33–3.19	1–4	

*Abbreviation*: *SD* Standard deviation

### Clinical efficacy

**Fig. 1:**
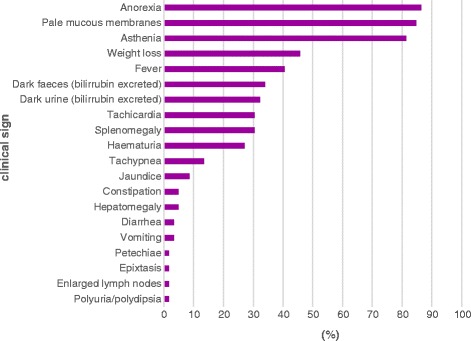
Percentage clinical signs observed on D0

Before treatment, mean body temperature was 38.94 °C (95% CI: 38.75–39.13) with a minimum of 36.5 °C and a maximum of 40.6 °C recorded. The most prevalent clinical signs observed in the physical examination were anorexia (86.4%), pale mucous membranes (84.7%), apathy (81.3%) and weariness (89.8%). Other reported clinical signs may be observed in Fig. 1.

**Fig. 2:**
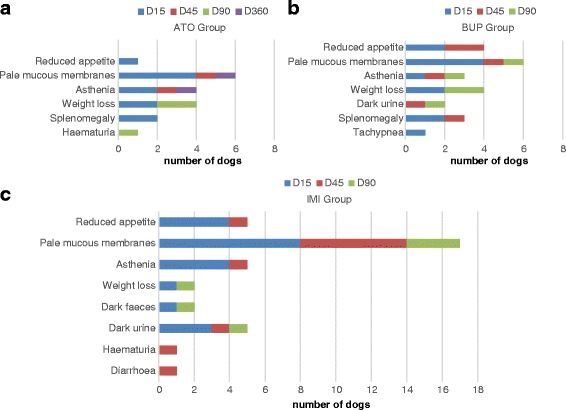
Number of dogs with clinical signs recorded during follow up. **a** Atovaquone treatment group (ATO). **b** Buparvaquone treatment group (BUP) **c** Imidocarb dipropionate treatment group (IMI)

On Day 15, 16/51 (31.4%) of dogs showed pale mucous membranes (8 of which were treated with IMI), 9/51 showed asthenia and 6/51 showed loss of appetite. The dogs showing worse clinical progression on Day 15 were those treated with IMI. On Day 45, pale mucous membranes were observed in eight dogs out of 17 (6 of which were treated with IMI), weariness in 5, apathy in 3 and appetite loss in 3. On Day 90, four dogs still had pale mucous membranes (Fig. 2).

**Fig. 3:**
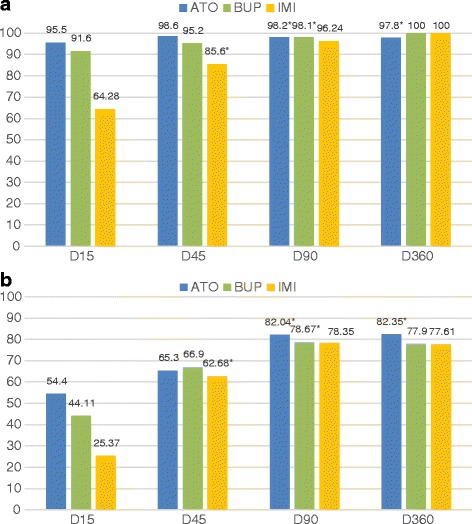
Percentage reductions recorded in clinical signs **a** and clinicopathological abnormalities **b** after treatment. *Asterisks indicate dogs showing a clinical relapse: 8 dogs on D45 in the IMI group, 2 dogs on D90 in the BUP group, and 3 dogs on D90 and 1 dog on D360 in the ATO group

No significant differences in scores were observed between different treatment groups (*F*_(2,39)_ = 0.179, *P* = 0.14). However, on Days 15, 45 and 90, clinical signs were reduced in greater measure in the groups ATO and BUP compared with IMI (Fig. 3a). After one year of follow up, all dogs showed a normal clinical status with the exception of one dog treated with ATO which presented with pale mucous membranes, weariness and apathy.

### Clinicopathological abnormalities

The main haematological finding before treatment was anaemia (93.22%; 55/59); in most of the dogs anaemia was regenerative (88.13%; 52/59) and in 5% (3/59) it was non-regenerative. Besides regenerative, anaemia was most often hypochromic and macrocytic. The second most prevalent finding was thrombocytopenia (71.4%; 30/42). Leukocyte counts were elevated in 27.1% (16/59) (mostly neutrophilia) and diminished in 2 dogs.

Biochemical abnormalities detected before treatment were elevated total serum proteins (17.5%; 10/57) and elevated hepatic enzyme activity (ALT) (10.7%; 6/56). Azotaemia was observed in 1.75% of cases (1/57) and elevated blood urea levels in 12.5% of the cases (7/56).

**Fig. 4:**
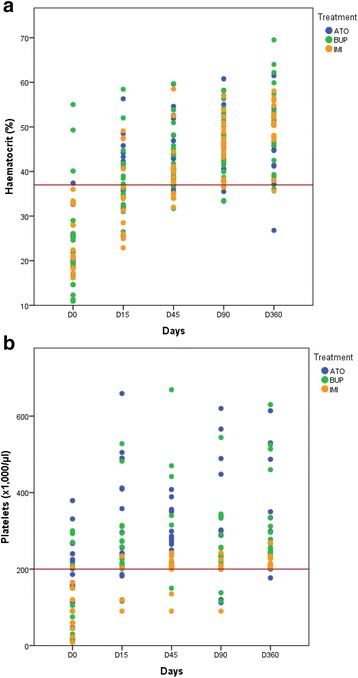
Haematocrit **a** and platelets counts **b** follow-up in treated dogs. The *red* lines indicate the reference values, i.e. 37% haematocrit **a** and 200 × 10^3^/μl platelets **b**

Following treatment, all three groups showed the similar behaviour of blood panel results. Red blood cell counts and haematocrits increased between each of the follow-up times (Greenhouse-Geisser, *G-G* = 99.6, *df* = 3.43, *P* ≤ 0.001; *G-G* = 104.7, *df* = 3.17, *P* ≤ 0.001; respectively) (Fig. 4a). A significant decrease was produced in MCV from Day 15 to Day 45 (Greenhouse-Geisser, *G-G* = 4.086, *df* = 3.12, *P* = 0.008) and values thereafter stabilized within the normality range (60–76 fl). Differences among the three groups in these three variables were not significant. Platelets counting were normalized on Day 15 in the ATO and BUP; and on D45 in the IMI group (Fig. 4b)

Leukocyte counts also behaved similarly over time in the three groups with significant reductions observed (Greenhouse-Geisser, *G-G* = 7.205, *df* = 2.764, *P* ≤ 0.001) from Day 0 to Day 15. Neutrophil counts also fell over time in the three treatment groups.

Total serum protein and creatinine levels also showed similar behaviour in the three groups with significant increases produced in the means of both variables relative to Day 0 for each time point (Greenhouse-Geisser, *G-G* = 5.3, *df* = 3.141, *P* = 0.02 and *G-G* = 19.609, *df* = 3.357, *P* ≤ 0.001, respectively).

**Fig. 5:**
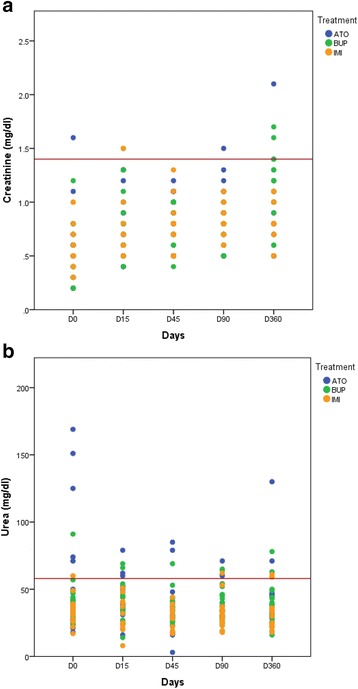
Creatinine **a** and urea **b** follow-up in treated dogs. The *red* lines indicate the reference values, i.e. 1.4 mg/dl creatinine **a** and 58 mg/dl urea **b**

At one year of follow up, 5.7% dogs had azotaemia (3/52) (Fig. 5a) and 12.7% elevated blood urea levels (6/47) (Fig. 5b) and elevated total serum protein levels were recorded in 29.4% of the dogs (15/51). The last nine dogs enrolled were also subjected to the SDMA kidney function test (7 in ATO, 2 in BUP). Of these 9 animals, 5 showed elevated SMDA levels on Day 90 (up to 14 μg/dl). However, only two of these five dogs (including the dog with high creatinine on Day 90) continued to show high SMDA levels (15 and 18 μg/dl) on Day 360.

The worst percentage reductions in clinicopathological abnormalities, between time points, were recorded in the dogs treated with IMI. On Day 15, dogs included in IMI group only showed a 25.4% reduction compared to 54.4 and 44.11% obtained in the ATO and BUP groups, respectively. Highest percentage reductions in laboratory abnormalities (82.3%) were observed on Day 360 in dogs treated with ATO (Fig. 3b).

### Blood smears

**Fig. 6:**
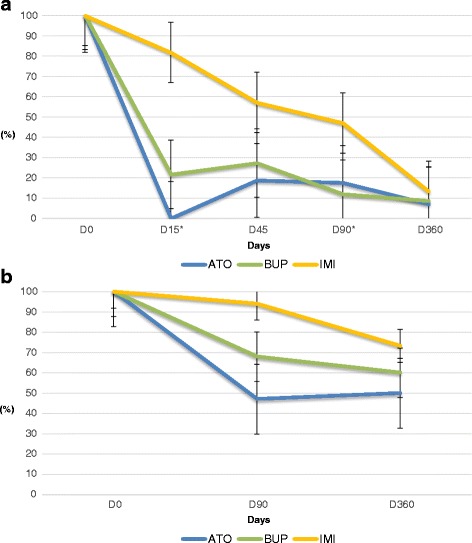
Percentages of dogs testing positive for the *Babesia microti*-like piroplasm infection dogs by blood smear **a** or PCR **b** before and after treatment. Asterisks indicate a significantly higher parasitaemia level in the IMI group *versus* ATO (*P* ≤ 0.001 on D15) and *versus* BUP (*P* = 0.001 on D15; *P* = 0.028 on D90)

Following treatment, the IMI group showed the largest number of dogs with blood smears testing positive for Bml infection (Fig. 6a). On Day 15, significant differences between groups were produced, whereby 9/11 dogs in IMI and 5/23 dogs in BUP showed positive blood smears *versus* no dogs in the ATO group (IMI *vs* ATO: *Z* = 20.86, *P* ≤ 0.001; IMI *vs* BUP: *Z* = -15.32, *P* = 0.001). Even by Day 45, more than half of the dogs in the IMI group tested positive (57.14%), while 18.7 and 27.27% were positive in ATO and BUP, respectively. On Day 90, significant differences in positive blood smears emerged between IMI and BUP (*Z* = -10.34, *P* = 0.028), the IMI group showing the highest percentage of dogs testing positive (47%) in comparison with the BUP group (12%). However, differences between ATO and BUP were not produced at this time point (Fig. 6a). After one year of treatment, 1/14 dog in ATO, 2/23 dogs in BUP and 2/15 dogs in IMI returned borderline positive blood smear results.

### Specific nested PCR

Significant differences in changes in PCR parasitaemia rates over time (Day 0 *vs* Day 90 and Day 360) were detected in the three groups (Greenhouse-Geisser, *G-G* = 18.266, *df* = 1.967, *P* < 0.001). After three months and 1 year of treatment, respectively, parasitaemia was PCR-confirmed in 47.05% (8/17) and 50% (7/14) of dogs treated with ATO, 68% (17/25) and 60.8% (14/23) of those treated with BUP and 94.11% (16/17) and 73.3% (11/15) of those treated with IMI (Fig. 6b). These differences between the three groups were not however significant (*F*_(2,49)_ = 2.057, *P* = 0.139).

### Safety and tolerance

No negative impacts on haematology or biochemistry variables (notably on renal or hepatic biomarkers) were recorded throughout the trial in the three treatment groups. In dogs in IMI, atropine (0.02 mg/kg SC) was used to avoid product-related cholinergic effects and there was pain upon injection on 26.6% of the cases, and 23.5% of dogs in the ATO group experienced vomiting lasting for 2–3 days after starting treatment, and omeprazol (0.5 mg /kg PO SID/10 d) given in those cases. The buparvaquone/azithromycin combination was well tolerated in all dogs and no product-related adverse events were reported.

### Relapses

We recorded 8/17 Bml-positive dogs by blood smear on Day 45 in the IMI group. These eight dogs showed clinical relapse indicated by a clinical score of 1 to 7 points. Six of these dogs presented with regenerative anaemia (mean 4.42 × 10^6^ cells/μl and haematocrit 36%) and two with thrombocytopenia. All were retreated with imidocarb dipropionate (one dose 5.5 mg/kg IM) at this time point.

Further dogs returning positive blood smears (3/25 in BUP and 3/17 in ATO on Day 90) showed clinical relapse: two dogs in BUP and three in ATO consisting of mild regenerative anaemia and clinical scores of 0 to 6 points. These six dogs were retreated with BUP or ATO at this time point.

## Discussion

Among many different babesicides used in veterinary medicine, imidocarb dipropionate and diminazene aceturate are the most widely used in cows and horses, while the former is the first choice of treatment for canine babesiosis caused by large *Babesia* species. In addition, the combination atovaquone/azithromycin has proved successful against some *Babesia* spp. that infect humans, particularly *Babesia microti* and *Babesia divergens* [7]. Several other piroplasms, including *B. gibsoni*, *B. conradae* and *Cytauxzoon felis* [11–13], respond to combined atovaquone/azithromycin treatment. In contrast, buparvaquone has been extensively tested for veterinary use against theileriosis in cattle [16] and babesiosis in horses [18], but there are no reports of its application in dogs and/or cats.

The present clinical trial was designed to compare the efficacy of three treatments (imidocarb dipropionate, IMI, atovacuone plus azithromycin, ATO and buparvaquone plus azithromycin, BUP) in an effort to improve the current clinical management of canine piroplasmosis caused by Bml. To our knowledge, this study is the first to compare the use of BUP against this small piroplasm in naturally infected dogs with that of ATO or IMI.

Out of 60 dogs included in our trial, 77.9% responded well to treatment in terms of clinical improvement while 22% dogs showed clinical relapse. Clinical signs present in several dogs at the time of inclusion were reduced in frequency and severity in response to treatment. These signs were: asthenia, decreased appetite, pale mucous membranes, fever, orangey faeces, pigmenturia, tachycardia, splenomegaly, haematuria, tachypnea and jaundice. Similar clinical signs have been reported in infected dogs with Bml in Spain [5, 29]. Overall, the disease course showed clinical improvement in response to treatment. Dogs showing the worst clinical progression on Days 15, 45 and 90 following treatment were those treated with IMI.

Similarly, main haematological abnormalities showed a general trend towards their reduced frequency and severity, including regenerative anaemia and thrombocytopenia, consistent with prior reports [5, 6]. Total leukocyte counts (mainly neutrophiles) also showed a decreasing tendency. Leukocyte disorders have been inconsistently observed in dogs with piroplasmosis [29]. This could be a consequence of the severe stress associated with this illness, in line with observations by other authors [5, 6].

In contrast, other clinicopathological alterations, such as total serum protein and creatinine levels remained elevated throughout the whole year of follow up. Thus, we recorded azotaemia rates of 1.7 and 5.7% at the start and end of follow up, respectively, and one dog died of kidney failure. Others have observed higher prevalence of kidney disease (36%) [6] in Bml infected dogs and suggested its strong correlation with likelihood of death within the first week of diagnosis [30]. In our study, the dogs that showed azotaemia at the start and end of follow up were not the same ones. Hence, azotaemia observed in dogs at the time of treatment (1.7%) responded well to treatment while 5.7% dogs, all of them hunting dogs, developed azotaemia after treatment. These latter animals may have developed immunomediated glomerulonephritis in the long term as a result of immune complex deposition. In a dog infected with *B. gibsoni*, Slode et al. (2011) reported membranoproliferative glomerulonephritis due to immune complex deposition [31]. However, it should be noted that most of the dogs in our study with Bml were hunting dogs [5, 29]. Thus, chronic kidney disease in these animals could be more associated with their poor management (including many hours without drinking while hunting over several days) than a proper consequence of Bml infection. There were some dogs with increased urea levels without azotaemia, it could be due to high protein food, hipertermia, and/or early prerenal azotaemia (shock, dehydration). More studies are needed using urine biomarkers of renal function from the early course of the infection to improve knowledge of the renal impacts of Bml infection in dogs.

Our study showed good correlation between LM and PCR on Day 0 (100% positive dogs, both methods), because all blood smears were observed by the same qualified technician. It should also be noted that we assume that most of the dogs examined in this study were in the clinical phase of Bml infection time-point when the visual detection of piroplasms is easier, than in animals with low parasitaemia levels due to chronic disease [5]. However, during the follow up (Day 90 and 360), PCR detected more positive dogs than LM in the three groups. In effect, LM has been described as less sensitive than PCR to detect chronic and subclinical piroplasmosis in carrier dogs [5, 32].

Generally, worse improvements in clinicopathological abnormalities at each time point (especially the first 45 days) were observed in the dogs treated with imidocarb dipropionate. Although this drug is the treatment of choice for canine babesiosis caused by large piroplasm species, in clinical cases caused by small *Babesia* species it seems incapable of completely eliminating parasites from the bloodstream at the recommended dose, only improving the severity of clinical signs [7]. Our study shows that IMI reduces parasitemia (both by blood smear and PCR) to a lesser extent than atovaquone or buparvaquone plus azithromycin, such that relapse was frequently seen and most dogs remained subclinically infected when treated with IMI. Treatment with ATO gave rise to better results in terms of clinical signs, clinicopathological abnormalities and parasitaemia. These results were similar to those obtained for BUP.

Atovaquone plus azithromycin has been used to treat small piroplasms such as *B. conradae* and *B. gibsoni* [12, 13]. None of twelve clinical cases of *B. conradae* treated with atovaquone plus azithromycin showed detectable *B. conradae* DNA by PCR at any time-point after treatment ended (Day 120) [13]. Similarly, *B. gibsoni* (Asian genotype) DNA was detectable by PCR in posttreatment samples in 2/10 treated dogs: one dog tested positive at 60 and 90 days post-treatment, and the other dog tested positive 90 days after treatment [12]. In the present study, the number of treated dogs was larger and follow up was longer than 120 days. On Day 15, Bml was not detected in blood smears but from Days 45 to 360, positive results were obtained, both by PCR and blood smear. The detection of dogs positive for the parasite after treatment indicates treatment failure, reinfection, or lack of adherence to treatment. The resistance of other protozoa to atovaquone has been associated with mutations in the cytochrome *b* gene [9, 33].

Buparvaquone plus azithromycin given at a dose of 5 mg/kg IM and a second dose 48 h later proved safe and was better tolerated than the other two treatment regimens. Some dogs treated with ATO experienced vomiting for two or three days after starting treatment. All dogs treated with IMI were first given atropine to avoid cholinergic effects. We observed a better clinical and parasitological efficacy of BUP than IMI suggesting buparvaquone could be an interesting new approach to treating small piroplasm infections in dogs. In effect, other authors have shown that buparvaquone, in combination with other drugs, could be a better choice than imidocarb against *B. equi* infection [18], though further clinical trials are required in horses.

While atovaquone monotherapy is not recommended for the treatment of human babesiosis caused by *B. microti,* its combination with azithromycin has proved effective in experimental animals and humans [9, 10]. Hence, it would be interesting to test the use of buparvaquone or atovaquone alone to treat Bml. This approach would limit the use of macrolides in animals and thus also helps minimize antibiotic resistance in humans.

So far, no specific safe and efficient treatment for piroplasmosis exists and the majority of dogs treated with specific antibabesial drugs are unlikely to be cured of their infection. Any dog with confirmed Bml infection should be regarded as potentially infected for life, despite specific treatment and remission of clinical signs [2]. The epidemiological role of dogs that continue subclinically infected after treatment remains unclear. Further clinical trials are needed to improve the clinical management of canine piroplasmosis in the small animal practice.

## Conclusions

Of the three treatments tested, imidocarb dipropionate showed the worse clinical and parasitological efficacy against *B. microti*-like piroplasm infection in dogs. We would therefore not recommend its use for this purpose. The combination atovaquone/buparvaquone plus azithromycin showed the best clinical and parasitological efficacy, though it was still incapable of completely eliminating the infection at the recommended dose. All three treatment regimens were well tolerated and safe and buparvaquone/azithromycin protocol was the only one not showing any adverse events in treated dogs.
